# Optimization of Green Extraction Techniques for Polyphenolics in *Pinus brutia* Bark Extract and Steam Gasification of the Remaining Fraction to Obtain Hydrogen-Rich Syngas and Activated Carbon

**DOI:** 10.1021/acsomega.4c01083

**Published:** 2024-12-13

**Authors:** Ece Yildiz-Ozturk, Pelin Secim-Karakaya, Fikret Muge Alptekin, Melih Soner Celiktas

**Affiliations:** † Department of Food Processing, Food Technology Programme, Yasar University, 35100 Bornova, Izmir, Turkey; ‡ Textile and Apparel Research-Application Center, Ege University, 35040 Bornova, Izmir, Turkey; § Ege University Solar Energy Institute, 35040 Bornova, Izmir, Turkey

## Abstract

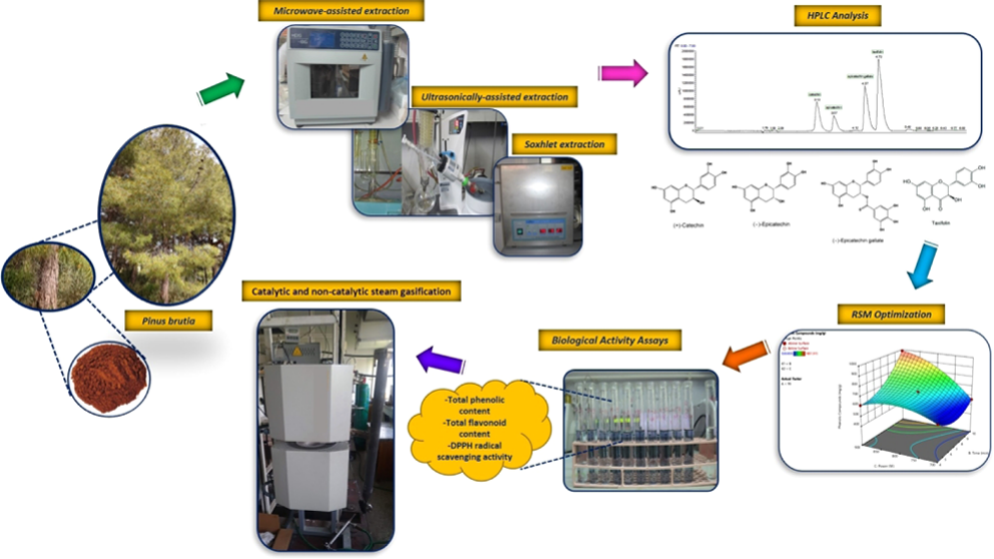

Utilization of renewable resources has become imperative, and considerable efforts have been devoted to tackling diverse global sustainability challenges, which contribute to the circular economy. The focus of this work was to optimize the extraction of polyphenolic compounds in *Pinus brutia* bark using microwave-assisted (MAE) and ultrasonically assisted (UAE) extractions and evaluate the biological efficacies of the extracts. Additionally, the residue of the extracted pine bark was subjected to steam gasification to produce hydrogen-rich syngas and activated carbon. The optimum process parameters for MAE were determined as 70 °C, 10 min, and 900 W, and 987.32 mg gallic acid equivalent (GAE), 23.7 mg quercetin/g extract, and 86.2% antioxidant activity were obtained. The optimum process parameters for UAE were determined as 70 °C, 20 min, and 50% power, and 811.84 mg gallic acid equivalent (GAE), 30.1 mg quercetin/g extract, and 90.8% antioxidant efficiency were obtained. The extracts obtained under optimized conditions were assessed for the bioactive phenolic compounds taxifolin, (−)-catechin, (−)-epicatechin, and (−)-epicatechin gallate by ultra performance liquid chromatography (UPLC). Especially in MAE (ethanol), taxifolin content was notable (34.0 mg/g extract), followed by UAE (ethanol) (23.5 mg/g extract). Compared to MAE (ethanol) and UAE (ethanol) with regards to catechin content, 1.05 mg/g extract and 0.81 mg/g extract were obtained, respectively. Catalytic and noncatalytic steam gasification of pine bark residue yielded 57.3 and 60.8 mol % H_2_, respectively. In addition, excellent tar reduction was achieved through utilizing a 10% boron-modified CaO alkali catalyst, and the obtained activated carbon exhibited 1358.32 m^2^/g Brunauer–Emmett–Teller (BET) surface area and 1.05 cm^3^/g total pore volume, which has potential use as an adsorbent for removing heavy metals and electrode material for supercapacitor application.

## 1 Introduction

The circular economy has gained importance in the last few decades intending to enhance resource value while minimizing waste through effective material management across the entire value chain.^1^ Biorefineries, when integrated into a circular bioeconomy framework, are poised to play a pivotal role in advancing sustainability.^2^ Biomass and biowastes have emerged as promising and renewable feedstocks for the development of biorefinery systems.^3^ Energy, bioactive compounds, chemicals, and fuels include solid, liquid, and gas products with different ratios and can be obtained via biorefinery by applying different methods.^4^ Recently, a variety of new modern extraction techniques have been employed to promote efficiency and extract quality, while decreasing extraction time and solvent consumption.^5^ The advanced green technologies currently used are microwave-assisted extraction (MAE),^6^ ultrasonically assisted extraction (UAE),^7^ supercritical fluid extraction,^8^ and pressurized solvent extraction.^9^ In order to enhance the recovery of extracts rich in bioactive substances from raw materials and preserve bioactivity, the process parameters for extraction should be optimized. Experimental design methods are used to optimize the extraction processes in order to improve the output value and reduce the costs.^10^ Apart from the extract phase, the remaining biomass is further processed to be utilized as biofuels,^11^ fibers,^12^ hydrogen,^13^ and carbon-based materials.^14^ Among these processes, gasification is one of the thermochemical conversion methods used to produce gas products from biomass. Based on the gasification conditions, the product ratio, including solids, liquids, and gases, can change.^15^ Steam, CO_2_, air, and mixtures of steam and air are used as gasification mediums.^16^ As for carbon-based materials, physical and chemical activation are the two main routes for specifically preparing activated carbon.^14^ Steam, serving as the gasification medium, can be used for the production of activated carbon through the physical activation method.

Europe has large forest areas, and the Eastern Mediterranean Basin is quite rich in red pine (*Pinus brutia*). The most productive and widespread areas of red pine in the world are in Turkey. It is referred to as “Turkish red pine” and is one of our important tree species with high economic value.

High amounts of forestry wastes are produced annually, and among them is pine, rich in terms of phenolic compounds such as phenolic acids 17, taxifolin, catechin, and epicatechin along with procyanidin and proanthocyanidins.^8,18^ Considering the sustainability and health impact in this context, the transformation of such forest wastes into potentially new products that can generate more income is of prime importance. Indeed, pine bark extracts are reported to exert anticancer, antidiabetic, antimicrobial,^7^ anti-inflammatory,^12^ antioxidative,^19,20^ and wound healing activities.^21^ Pine bark extracts have been successfully applied on solid surfaces,^22^ such as cotton fabrics to prevent bacterial infection and accelerate wound healing,^23^ especially by increasing skin keratinocyte cell proliferation.^24^ Within the context of this study, we optimized the process parameters of MAE and UAE maximizing the phenolic content using response surface methodology (RSM). The taxifolin, catechin, epicatechin, and epicatechin gallate constituents of the extracts were quantified with ultra performance liquid chromatography (UPLC) and antioxidant activities were determined. Additionally, the residue of the extracted pine bark was subjected to steam gasification to produce hydrogen-rich syngas and activated carbon. Based on the literature examination, there is no gasification study that was carried out using steam as a gasifying agent. In this context, catalytic and noncatalytic steam gasification of the remaining pine bark extract was performed to obtain hydrogen-rich syngas and activated carbon. The gas products and activated carbons obtained were then evaluated in terms of their gas composition (mol %) and specific surface area-porous structure, respectively.

## 2 Materials and Methods

### 2.1 Plant Material

*P. brutia* bark was picked up from Izmir-Deliomer village (38°10′01″N 27°03′35″E) between June and August in Turkey in 2020. The samples were dehydrated at room temperature and ground in a lab-scale commercial blender. It was then stored at +4 °C until extraction.

### 2.2 Materials and Reagents

Folin–Ciocalteu’s reagent, 2,2-diphenyl-1-picrylhydrazyl hydrate (DPPH), aluminum chloride (AlCl_3_), and sodium carbonate (Na_2_CO_3_) were supplied by Sigma. All solvents for extraction and UPLC analysis were purchased from Merck.

The standards used for the determination of flavonoids and phenolic acids, namely taxifolin, (−)-catechin, (−)-epicatechin gallate, and (−)-epicatechin were purchased from Sigma-Aldrich.

### 2.3 Extraction Processes of the *P. brutia* Bark

#### 2.3.1 Microwave-Assisted Extraction

Microwave-assisted extraction experiments were performed using microwave equipment (Sineo Microwave Chemistry Technology Co., Shanghai) containing eight cartridges. About 2 g of dried and ground pine bark were mixed in water and ethyl acetate (solid/liquid ratio: 1/5) and placed in cartridges. MAEs were carried out according to the Box–Behnken experimental design with the independent variables of temperature (40, 70, 100 °C), extraction time (4, 7, 10 min), and power (700, 800, 900 W). At the end of each extraction process, it was allowed to stand for 5 min for the device to cool down, and the samples were taken out of the cartridges. The mixtures were filtered with filter papers to separate the resulting liquid phase, which was evaporated in a rotary vacuum evaporator and then stored at +4 °C until biological analysis.

#### 2.3.2 Ultrasonically Assisted Extraction

Ultrasonically assisted extractions were performed with an ultrasonic bath (Everest Ultrasonic, Istanbul, Turkey). About 2 g of dried and ground pine bark was mixed in water and ethyl acetate (solid/liquid ratio: 1/5) and placed in centrifuge tubes. UAEs were carried out according to the Box–Behnken experimental design with the independent variables of temperature (40, 70, 100 °C), time (20, 40, 60 min), and power (50, 75, 100%). Then, the obtained mixtures were filtered with filter paper, and the separated liquid phase was evaporated in a rotary vacuum evaporator and then stored at +4 °C until biological analysis.

#### 2.3.3 Soxhlet Extraction

Soxhlet extraction was carried out using a 500 mL Soxhlet apparatus. About 50 g of dried and ground pine bark were placed in cartridges prepared with filter papers, placed in the Soxhlet apparatus, and extracted with a water/ethanol mixture. Under reflux, the flush cycle was performed 4 times and the process was terminated. The solvent content of the extracts obtained was evaporated in a rotary vacuum evaporator and then stored at +4 °C until biological analysis.

#### 2.3.4 Extraction Yield

The extraction yield was calculated according to the formula
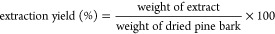


### 2.4 UPLC Analysis of *P. brutia* Bark Extracts

#### 2.4.1 Sample Preparation

The obtained extracts were dissolved in 50% aqueous methanol with a final concentration of 5 mg/mL to detect the taxifolin (Fluka, Steinheim, Russian Fed), (−)-catechin (Sigma), (−)-epicatechin (Sigma), and (−)-catechin gallate (Sigma) of the raw material.

#### 2.4.2 UPLC-UV Conditions

Ultra performance liquid chromatography (UPLC) analyses were executed on a UPLC device equipped with Thermo Scientific UPLC (Accela 1250 pump, Accela PDA detector, Accela Autosampler) using a Hypersil ODS-2 (4.6 × 150 × 3 μm^3^; Sigma Aldrich) column. The mobile phase consists of a mixture of 2% acetic acid (A) with water and acetonitrile (B). Gradient elution was carried out starting with 90A/10B, changing the composition to 60A/40B in 5 min, followed by 10A/90B in 7 min. The wavelength used for the measurement was 200–400 nm, the flow rate was 1 mL/min, the sample injection amount was 1000 μL/min, and the column ambient temperature was 30 °C.

#### 2.4.3 Calibration

Calibration curves of 5 mg taxifolin (Fluka, Steinheim, Russian Federation), (+)-catechin (Sigma), (+)-epicatechin (Sigma), and (+)-catechin gallate (Sigma) 5 mL in methanol followed by serial dilution of the stock solutions with methanol in the range of 1000–0.3 μg/mL. All standard samples were injected at 10 μL. The detector response was linear (*R*^2^ ≥ 0.9988) over the injected concentration range, with the detection limit less than 0.05 μg/mL. Analyses were performed in triplicate, and a “linear” calibration curve was determined using the Thermo Scientific Surveyer Chromquest 4.2 software.

#### 2.4.4 Experimental Design and Response Surface Methodology

The response surface methodology (RSM) is an experimental statistical technique that also includes an optimization study. Parameters influencing the process are called independent variables, and dependent variables are called responses. RSM can create a mathematical model and allow us to examine possible relationships between test variables while minimizing the number of experiments.^10^ The efficiency of extraction process parameters such as temperature, time, and power on the extraction of polyphenolic components from *P. brutia* bark by MAE and UAE methods was investigated by Box–Behnken design (BBD) using RSM, and optimum conditions were determined. The lower and upper limits of the independent variables were ascertained by preliminary experiments and a factorial design with three levels, three different temperatures, three different times, and three different powers were used, where extraction yield and total phenolic content were the responses. Optimization was performed using the Design-Expert (Design-Expert 13.0.0) statistical program. The preciseness of the model was evaluated according to the lack of fit, coefficient of fit (*R*^2^), and Fisher test value (*F*-value), which are the analysis of variance (ANOVA) outputs of the program. Models with a regression rate above 95% are acceptable. In this case, the inconsistency between the experimental data and predicted data is less than 5%. Other parameter is that the *p*-value implemented for the chosen model is less than 0.05, which points out that the model terms are significant. When a factor’s *p*-value is less than 0.05, it significantly affects the process for a confidence level of 0.95.^10,25^ The total phenolic component amount and extraction yield responses obtained together with the independent and experimental variables are given in Tables 1 and 3.

**Table 1:** Extraction Yield (%) and Total Phenolic Contents (mg/g) of *P. brutia* Extracts Obtained by MAE

exp. no	temperature (°C)	time (min)	power (W)	extraction yield (%)	total phenolics (mg/g)
1	40	10	800	6.87	761.099
2	70	10	900	5.19	987.315
3	100	7	900	10.65	530.655
4	40	7	900	9.11	678.647
5	70	4	700	12.98	568.710
6	100	10	800	12.10	625.793
7	100	4	800	7.03	911.205
8	40	7	700	9.53	562.368
9	70	7	800	9.02	649.049
10	70	10	700	15.37	558.140
11	70	4	900	12.29	613.108
12	40	4	800	7.62	752.643
13	70	7	800	8.02	695.560
14	100	7	700	13.02	553.911
15	70	7	800	9.24	654.939

### 2.5 Biological Activity Assays

#### 2.5.1 Total Phenol Content Assay

The total amount of phenolic compounds in *P. brutia* extracts was determined by the Folin–Ciocalteu method using gallic acid as the standard. The obtained extracts were diluted with water to 5 mg/mL. 100 μL of extract and 500 μL of Folin reagent were mixed, and then 1.5 mL of 20% Na_2_CO_3_ solution was added and left at room temperature for 1 h. The absorbance of the obtained mixtures was quantified at 760 nm with a UV-2401 (Shimadzu) spectrophotometer. The total amount of phenolics is presented as mg gallic acid equivalents (GAE) per gram of extract. Standard (gallic acid) curve equation: 760 nm = 0.0946·*c*_gallic acid_ (mg/mL).^5^

#### 2.5.2 Antioxidant Activity Assay

The antioxidant capacity of pine bark extracts was determined by the 2,2-diphenyl-1-picrylhydrazyl (DPPH) radical scavenging activity method, which is based on the scavenging of the DPPH radical by the redox reaction of antioxidant compounds. The extracts obtained according to this method were dissolved in 4 mL of methanol to a final concentration of 250 μg/mL. Then, 0.5 mL of DPPH (Sigma) (1 mM) methanolic solution was added to the extracts, mixed, and left in the dark at room temperature for 30 min. The absorbances of the obtained mixtures were quantified at 517 nm with a UV-2401 (Shimadzu) spectrophotometer.^12^ The low absorbance value obtained means a high free radical scavenging ability.

The antioxidant activity is given as percent inhibition and is calculated according to the following equation
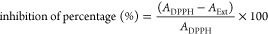


where *A*_DPPH_ is the absorbance value of DPPH solution for control and *A*_Ext_ is the absorbance value of the extract sample.

#### 2.5.3 Total Flavonoid Content Assay

Total flavonoid contents of *P. brutia* extracts were evaluated by the aluminum chloride colorimetric method, which is based on the formation of the Al^3+^ complex (yellow color) when flavonoids react with Al^3+^. Five hundred microliters of extract (1 mg/mL in MeOH), 1.5 mL of MeOH, 0.1 mL of 10% AlCl_3_ solution, 0.1 mL of CH_3_COOK (potassium acetate) aqueous solution (1 M), and 2.8 mL of distilled water were incubated at room temperature for 30 min. The absorbance of the mixture was then measured at 415 nm with a UV-2401 (Shimadzu) spectrophotometer. The amount of flavonoid content is shown as mg quercetin (mg QE/g) per gram of dry extract [calibration curve: 415 nm = 0.0037·*c*_quercetin_ (mg/g) + 0.0027].^26^

### 2.6 Catalytic and Noncatalytic Gasification of Pine Bark Residue

**Figure 1:**
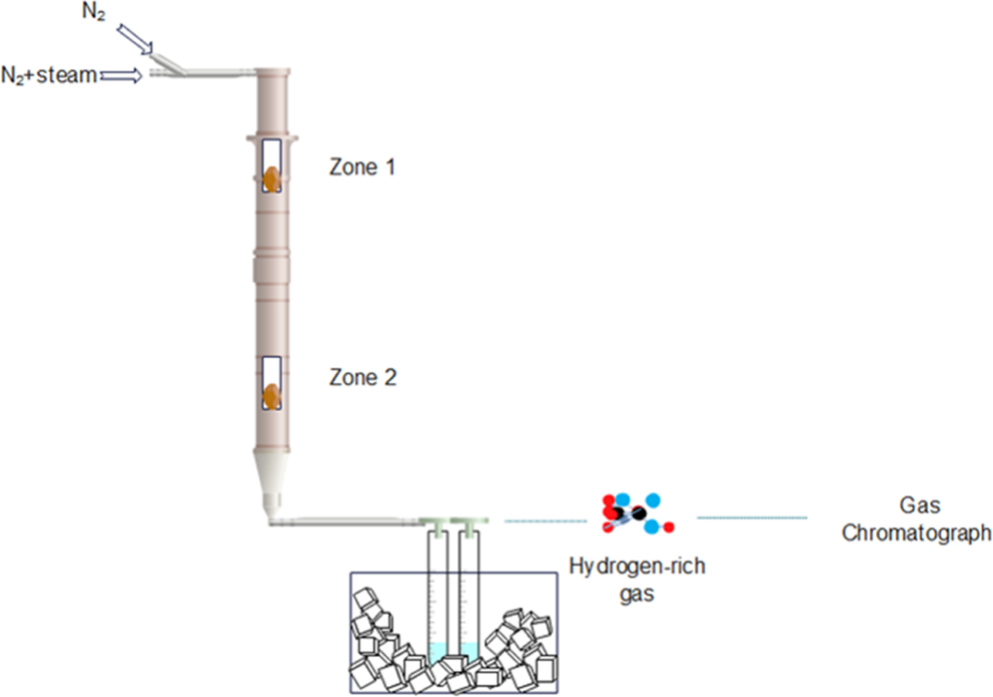
Schematic illustration of the gasification system.

Steam gasification studies were performed in two sections of electrically heated tubular furnaces (zones 1 and 2). The temperature in each segment, which has its own PID controller, may be controlled independently.^27^ The schematic illustration of the gasification system is shown in Figure 1. During a conventional noncatalytic experiment, the specimen was positioned within a stainless-steel receptacle suspended from an elevated metal rod within the reactor, specifically in zone 2. When zone 2 reached 200 °C, produced gas began to collect. Gasification experiments were conducted in permanent steam and nitrogen conditions at 850 °C for 1 h. Continuous streams of nitrogen and steam were maintained at a steady flow rate of 50 mL/min each from the reactor.

For catalytic gasification, a catalytic system incorporating a 10% boron-modified CaO alkali catalyst was employed. In a standard catalytically augmented experiment, the specimen and catalyst were situated within a stainless-steel receptacle suspended from distinct metal rods at the top (zone 1) and bottom (zone 2) of the reactor, respectively. First, zone 2 which contains the catalyst was heated to 850 °C, and then zone 1 began to heat up. Zone 1 was at 200 °C, and gas was beginning to collect. When it reached 850 °C, gasification experiments were conducted by maintaining constant conditions of steam and nitrogen at a temperature of 850 °C for 1 h. The biomass-to-catalyst ratio was kept at 1:1. The reactor ensured a continuous flow of nitrogen and steam at rates of 50 mL/min each.

Both noncatalytic and catalytic gasification experiments were carried out using 5 g of pine bark-extracted residue. Produced syngas was collected in Tedlar gas sampling bags and analyzed by using a gas chromatograph (Agilent 7890A). In gasification experiments, generated tar was collected. After the completion of gasification experiments, solid products were meticulously gathered and categorized as PB and PB-B. These classifications correspond to distinct gasification conditions, specifically those involving noncatalytic and catalytic processes. The surface area of the resulting carbon was assessed through the Brunauer–Emmett–Teller (BET) method, involving the analysis of N_2_ adsorption–desorption isotherms at a temperature of −196 °C (Micromeritics, Gemini VII 2390t). The Barrett–Joyner–Halenda (BJH) method was employed to derive the pore size distribution (PSD) from the desorption branch of the isotherm.

## 3 Results and Discussion

### 3.1 Optimization of Polyphenolic Compound Extraction by MAE

Microwave-assisted extraction (MAE) is influenced by various variables such as irradiation time, intensity, temperature, solvent type, and the interactions of all of these factors. For this reason, it is necessary to determine the optimum extraction conditions with statistical optimization. A quadratic polynomial equation was obtained to denote the extraction yields (%) and total phenolics (mg/g extract *P. brutia*) as a function of independent variables. In order to determine the significant effects of process variables, variance analysis (ANOVA) (*p* < 0.05), comparing the two levels of a single factor, the tested and the proposed extraction results, was performed and the most suitable model was determined. The regression coefficients of the proposed experimental model were investigated for each response, the statistical significance of all parameters was determined, and the significant and nonsignificant effects in the model were evaluated (Table 1).

**Figure 2:**
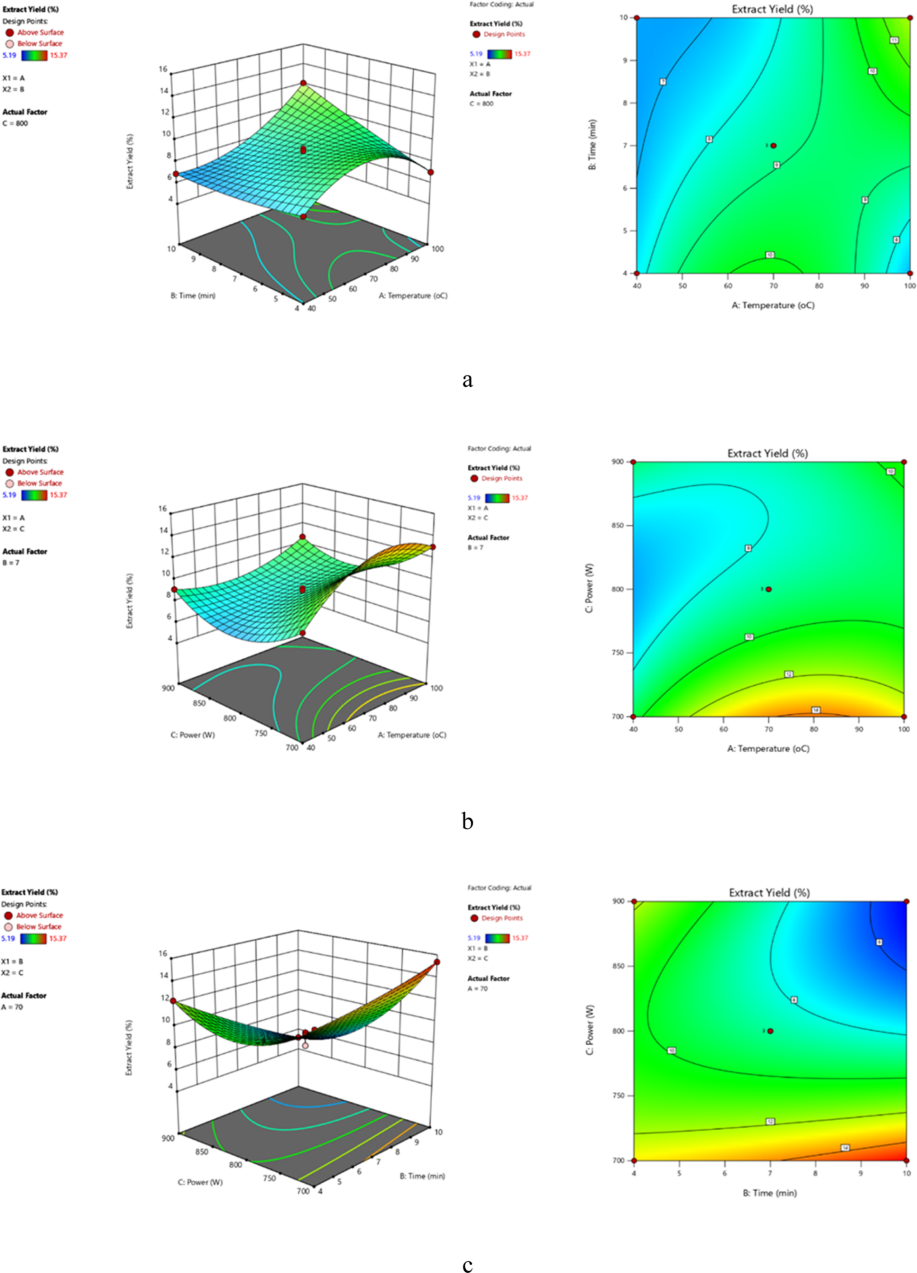
Three-dimensional (3D) and contour response surface plots of MAE yield showing the effects of temperature and time at a constant optimum power (800 W) (a), the effect of temperature and power at a constant time (7 min) (b), and the effect of time and power at constant temperature (70 °C) (c).

The statistical experimental design was applied to characterize the impacts of temperature (40–100 °C), extraction time (4–10 min), and power (700–900 W) on the extraction yield and total phenolic content of *P. brutia* bark and their interactions (Figure 2). The regression coefficients of the linear, quadratic, intersection, and interaction terms of the experimental model were computed utilizing the least-squares technique. Model equivalence was generated for all factor levels, and the degree of influence of each variable on the extraction of polyphenolic components was calculated. Correlations between independent variables were computed and evaluated with the first- and second-order coefficients of polynomial equivalences. Based on ANOVA variance analysis, the fitted cubic polynomial model part data are given with high-resolution available correlation coefficients (*R*^2^), 0.9924 and 0.9948 for extraction yield and total phenolics. The high *R*^2^ value indicates that there is good agreement between the experimental results and the theoretical results obtained from the proposed model. It shows that 99.5% of the variations in MAE of pine bark polyphenols were attributed to independent variables and only 0.5% could not be explained by the model. However, a high *R*^2^ value does not always indicate the fit of the regression model. In a good fit model, Adj-*R*^2^ should be comparable to *R*^2^. As shown in Table 2, the model *R*^2^ and Adj-*R*^2^ values are close to each other. The statistical significance of the model (*p*: 0.0450* < 0.05 and *p*: 0.0307* < 0.05) indicates the effectiveness of the model equation in estimating the return under any combination in terms of the values of the variables.

**Table 2:** Analyses of Variance (ANOVA) for the Fitted Cubic Polynomial Models for Microwave-Assisted Extraction (MAE)

source	sum of squares	df	mean square	*F*-value	probability (*p*) > *F*
extraction yield					
model	109.70	12	9.14	21.62	0.0450
*A*—temperature	6.33	1	6.33	14.96	0.0608
*B*—time	5.55	1	5.55	13.12	0.0685
*C*—power	29.54	1	29.54	69.87	0.0140
*AB*	8.47	1	8.47	20.03	0.0465
*AC*	0.9506	1	0.9506	2.25	0.2725
*BC*	22.52	1	22.52	53.25	0.0183
*A*^2^	1.41	1	1.41	3.33	0.2096
*B*^2^	0.2544	1	0.2544	0.6018	0.5191
*C*^2^	21.89	1	21.89	51.78	0.0188
pure error	0.8456	2	0.4228		
cor total	110.55	14			
*R*^2^	0.9924				
*R*^2^ adj	0.9465				
adeq precision	16.817				
total phenolics					
model	2.46 × 10^–5^	12	20 530.17	32.01	0.0307
*A*—temperature	6119.01	1	6119.01	9.54	0.0908
*B*—time	33 057.85	1	33 057.85	51.54	0.0189
*C*—power	56 067.83	1	56 067.83	87.42	0.0112
*AB*	21 589.74	1	21 589.74	33.66	0.0284
*AC*	4867.50	1	4867.50	7.59	0.1104
*BC*	37 013.53	1	37 013.53	57.71	0.0169
*A*^2^	16.70	1	16.70	0.026	0.8866
*B*^2^	35 675.41	1	35 675.41	55.63	0.0175
*C*^2^	25 432.45	1	25 432.45	39.65	0.0243
pure error	1282.70	2	641.35		
cor total	2.476 × 10^–5^	14			
*R*^2^	0.9948				
*R*^2^ adj	0.9637				
adeq precision	19.369				

**Figure 3:**
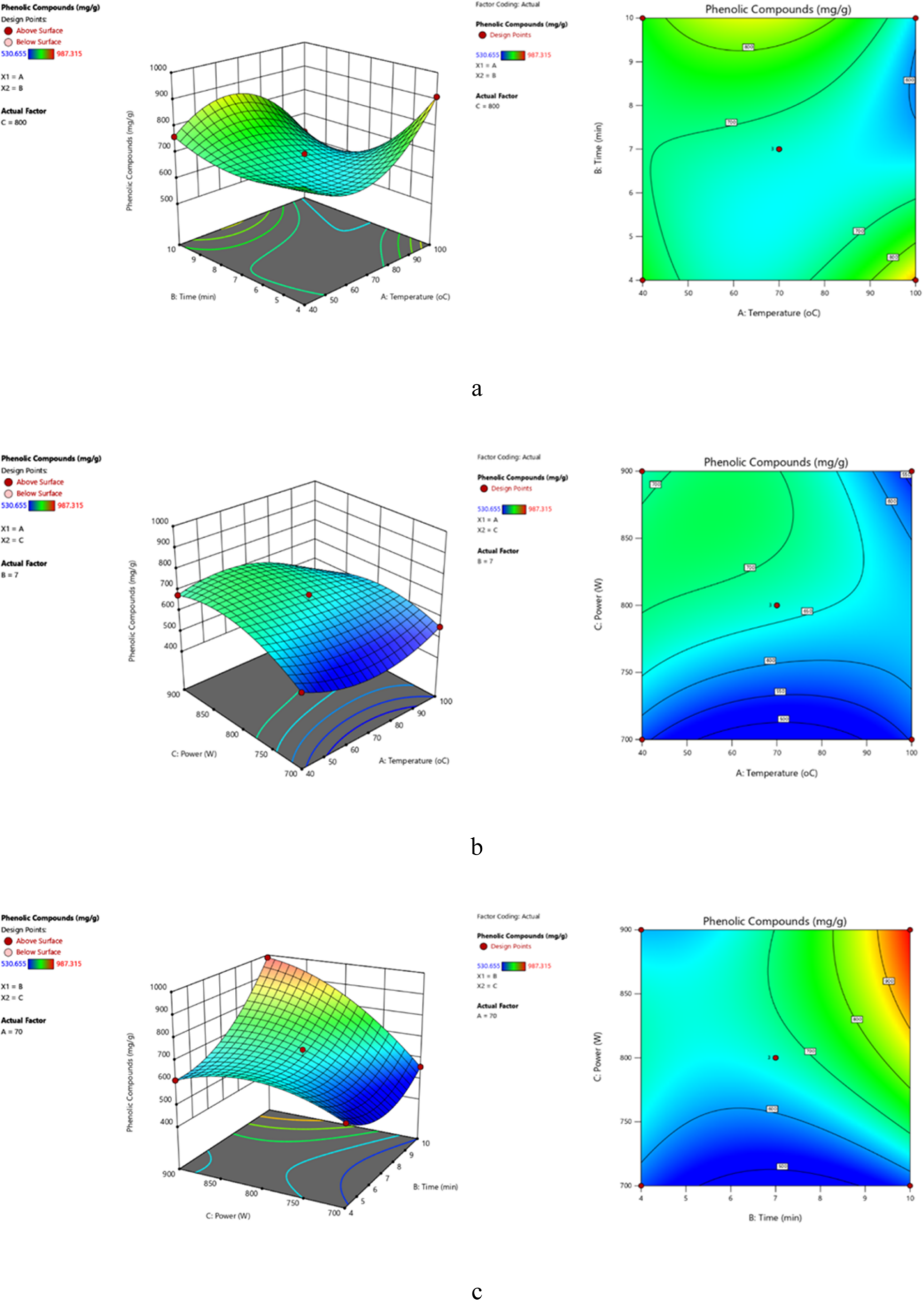
Three-dimensional and contour response surface plots of total phenolic compounds depicting the effects of temperature and time at a constant optimum power (800 W) (a), the effect of temperature and power at a constant time (7 min) (b), and the effect of time and power at constant temperature (70 °C) (c) in MAE.

While power (*C*) (*p* < 0.05) was found to be significant, temperature (*A*) and time (*B*) (*p* > 0.05) were not statistically significant for extraction yield. Additionally, temperature–time (*AB*) and time–power (*BC*) interactions (*p* < 0.05) were found to be significant, while temperature–power (*AC*) interactions (*p* > 0.05) were not found to be significant. While time (*B*) and power (*C*) (*p* < 0.05) were significant for phenolic compounds, temperature (*A*) (*p* > 0.05) was not statistically significant. In addition, temperature–time (*AB*) and time–power (*BC*) interactions (*p* < 0.05) were significant, whereas temperature–power (*AC*) (*p* > 0.05) interactions were not found to be significant (Table 2). Three-dimensional response surface profiles of multiple nonlinear regression models were drawn to investigate the interactive effects of independent variables and their effectiveness on the phenolic compounds, allowing the analysis of interactions between any two parameters and effectively position the optimum range of parameters, thus maximizing the overall phenolic content. When the effect of temperature and time on phenolic compounds at constant microwave power value (midpoint for power: 800 W) was examined, it was noted that both the maximum extraction yield and phenolic content were attained at 100 °C and 10 min (Figures 2a and 3a). When the effect of microwave temperature and power on phenolic compounds was examined at a fixed extraction time (midpoint for extraction time: 7 min), it was observed that the yield was maximized at 100 °C and 700 W power, whereas the phenolic compounds were maximized at 70 °C and 800 W power (Figures 2b and 3b). When the effect of extraction time and power on phenolic compounds was examined at constant extraction temperature (midpoint for extraction temperature: 70 °C), it was examined that yield was maximized at 10 min and 700 W power, whereas the phenolic compounds were maximized at 7 min and 800 W power (Figures 2c and 3c).

The quadratic polynomial equations for estimating the extraction yield and total phenolic content as a result of the regression analysis are shown below. In the equations, *A* is shown as temperature (°C), *B* as time (min), and *C* as power (W).
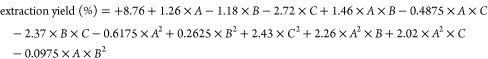

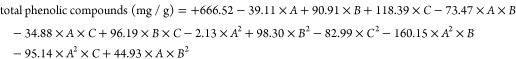


The efficiency of MAE of a herbal raw material depends on the microwave irradiation power, the structure of the herbal matrix, and the type of solvent.^28^ Microwave power and extraction time are important process parameters that affect the yield of bioactive compounds. The power of the microwave should be adequate to avoid the evaporation of the solvent used and prevent the deterioration of bioactive components and depletion of volatile substances.^29^ Utilizing less than 900 W of power during the 10-min extraction period resulted in a lower microwave energy in the herbal extract. A microwave power of 700–900 W applied for 10 min maximized the extraction efficiency and amount of phenolic compounds by enhancing the breaking of matrix–analyte bonds.

Microwave irradiation plays a critical role in the degradation of plant cell walls. In addition, microwaves affect the physical structure of the cell with the dipole moment, molecular movement, and rotation of the solvent used and facilitate the passage of the solvent into the cell by heating up quickly. Unlike traditional extraction processes based on the solubilization process, bioactive components can be removed from the sample matrix by increasing the solvent temperature and internal pressure in MAE.^30^ Microwaves can easily penetrate plant tissues and create thermal effects due to high frequencies, leading to the breakdown of the cell walls and allowing the intracellular fluids to interact with the solvents used to obtain much more bioactive components. The heating rate in microwave processes is 10–100 times faster than that in conventional heating methods. Therefore, MAE provides ideal conditions for the efficient extraction of bioactive components.^31^ The high bioactive compound yield of MAE in short processing times makes this technique advantageous compared to other methods. With the use of sufficient solvent to minimize energy consumption, temperature, time, and power parameters can be adjusted to obtain desired values. The environmentally friendly feature of the extraction process is the basis for potential large-scale application of MAE.^32^

In addition to the experiments conducted, a final MAE was carried out under the optimized conditions in order to verify the predictive capacity of the model. According to the Box–Behnken experimental design, the optimum extraction conditions for extraction yield in the MAE were determined as 88 °C, 9 min, and 704 W, and 16.16% extraction efficiency was obtained under these conditions. The highest extraction yield obtained under the experimental operating conditions of 70 °C, 10 min, and 700 W extraction was determined as 15.37%. As such, the optimum extraction conditions for total phenolics were determined as 62 °C, 10 min, and 895 W in MAE and 991.605 mg/g polyphenolic compound was obtained under these conditions. The highest phenolic content obtained under the operating conditions of 70 °C, 10 min, and 900 W was determined as 987.315 mg/g phenolic component. The results showed that there was no significant difference (*p* > 0.05) between the predicted and experimental values, which supports the compatibility of the experiments with the optimum stage and the suitability of the created models.

### 3.2 Optimization of Polyphenolic Compound Extraction by UAE

Ultrasonically assisted extraction (UAE) is affected by many factors such as time, intensity of impact, temperature, solvent type, and interactions of all of these factors. Thus, it is crucial to elicit the optimum process conditions. A quadratic polynomial equation was obtained to denote the extraction yields (%) and total phenolics (mg/g extract *P. brutia*) as a function of independent variables (Table 3).

**Table 3:** Extraction Yield (%) and Total Phenolic Contents of *P. brutia* Bark Extracts Obtained by UAE

exp. no	temperature (°C)	time (min)	power (%)	extraction yield (%)	total phenolics (mg gallic acid equivalent/g extract)
1	100	40	50	13.25	287.526
2	70	40	75	12.69	627.696
3	100	40	100	10.12	205.074
4	100	20	75	11.93	336.152
5	70	40	75	13.20	589.852
6	40	40	100	12.02	429.175
7	40	40	50	12.15	450.317
8	70	60	50	12.21	446.089
9	70	20	100	12.31	520.085
10	40	60	75	12.42	509.514
11	70	40	75	13.20	659.619
12	70	20	50	15.20	811.839
13	40	20	75	12.83	467.230
14	100	60	75	12.43	344.207
15	70	60	100	11.81	319.239

The experiments were carried out according to the generated experimental design to characterize the effects of temperature (40–100 °C), time (20–60 min), and power (50–100%) on the extraction yield and total phenolic content (Figure 3). Model equivalence was generated for all factor levels, and the degree of influence of each variable on UAE of phenolic compounds from pine bark was calculated. Correlations between independent variables were computed and evaluated with the first and second-order coefficients of polynomial equivalences (Table 4). Based on ANOVA variance analysis, the fitted cubic polynomial model data is given with high-resolution available correlation coefficients (*R*^2^), 0.9917 and 0.9932 for extraction yield and total phenolics. As shown in Table 4, model *R*^2^ and Adj-*R*^2^ values are close to each other, indicating that they are good statistical models. The statistical significance of the model (*p*: 0.0489* < 0.05 and *p*: 0.0401* < 0.05) indicates the effectiveness of the model equation in estimating the return under any combination in terms of the values of the parameters.

**Table 4:** Analyses of Variance (ANOVA) for the Fitted Cubic Polynomial Models for UAE

source	sum of squares	df	mean square	*F*-value	probability (*p*) > *F*
extraction yield					
model	15.51	12	1.29	19.87	0.0489
*A*—temperature	0.1600	1	0.1600	2.46	0.2573
*B*—time	3.05	1	3.05	46.82	0.0207
*C*—power	2.71	1	2.71	41.61	0.0232
*AB*	0.2070	1	0.2070	3.18	0.2163
*AC*	2.25	1	2.25	34.60	0.0277
*BC*	1.55	1	1.55	23.83	0.0395
*A*^2^	2.19	1	2.19	33.74	0.0284
*B*^2^	0.1897	1	0.1897	2.92	0.2298
*C*^2^	0.3123	1	0.3123	4.80	0.1598
pure error	0.1301	2	0.0650		
cor total	15.64	14			
*R*^2^	0.9917				
*R*^2^ adj	0.9418				
adeq precision	21.398				
total phenolics					
model	3.561 × 10^–5^	12	29 676.07	24.33	0.0401
*A*—temperature	37 421.39	1	37 421.39	30.68	0.0311
*B*—time	80 257.81	1	80 257.81	65.80	0.0149
*C*—power	43 807.46	1	43 807.46	35.91	0.0267
*AB*	292.90	1	292.90	0.24	0.6726
*AC*	939.75	1	939.75	0.77	0.4727
*BC*	6798.40	1	6798.40	5.57	0.1421
*A*^2^	1.424 × 10^–5^	1	1.424 × 10^–5^	116.72	0.0085
*B*^2^	839.49	1	839.49	0.69	0.4940
*C*^2^	27 518.98	1	27 518.98	22.56	0.0416
pure error	2439.59	2	1219.80		
cor total	3.586 × 10^–5^	14			
*R*^2^	0.9932				
*R*^2^ adj	0.9524				
adeq precision	18.662				

While time (*B*) and power (*C*) (*p* < 0.05) were found to be significant, temperature (*A*) (*p* > 0.05) was not statistically significant for extraction yield. Additionally, temperature–power (*AC*) and time–power (*BC*) interactions (*p* < 0.05) were found to be significant, while temperature–time (*AB*) (*p* > 0.05) interactions were not found to be significant. On the other hand, extraction temperature (*A*), time (*B*), ultrasound power (*C*) (*p* < 0.05) were found to be statistically significant for phenolic compounds. However, the interactions of temperature–time (*AB*), temperature–power (*AC*) and time–power (*BC*) (*p* > 0.05) were not found to be significant (Table 4).

**Figure 4:**
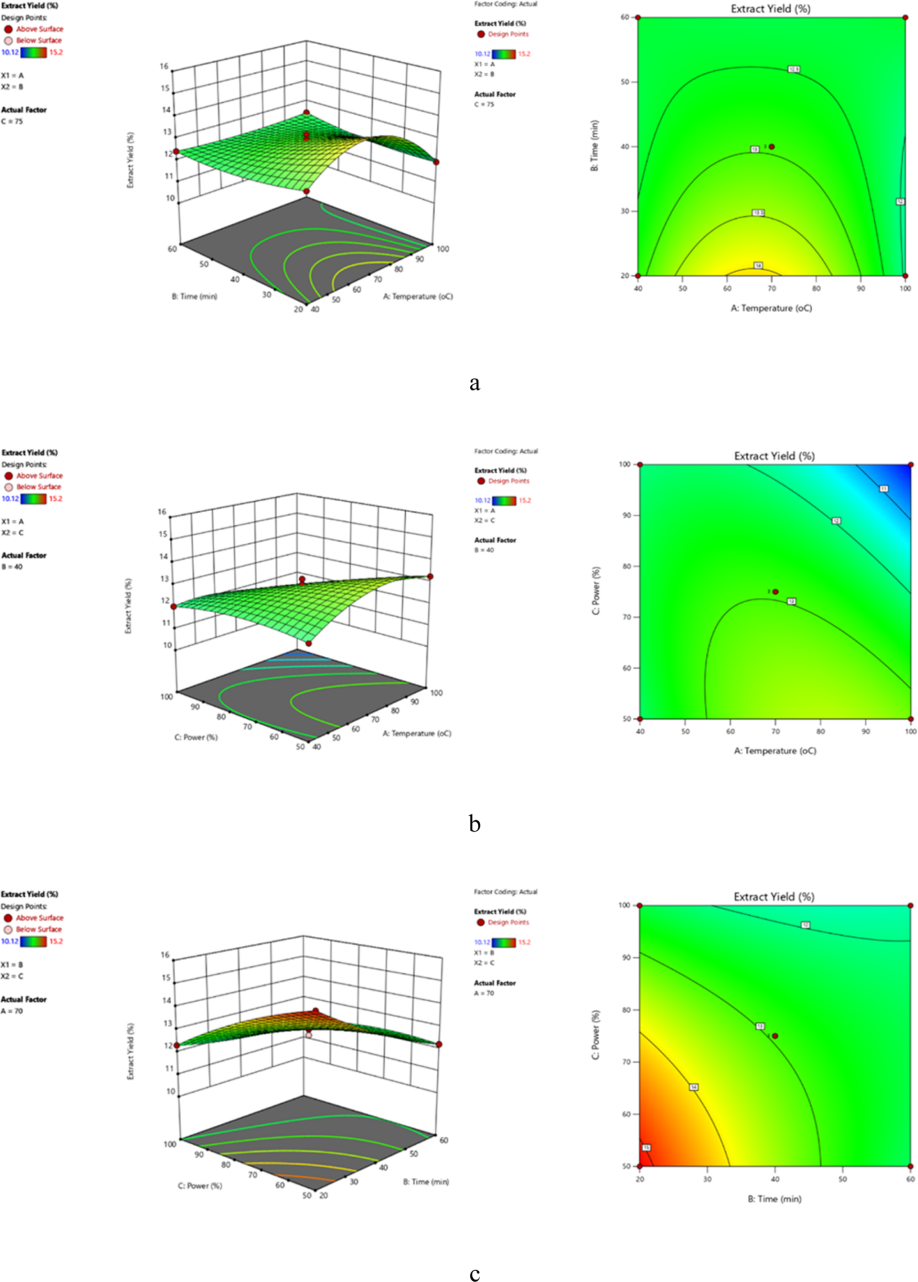
Three-dimensional and contour response surface plots of UAE yield showing the effects of temperature and time at a constant optimum power (800 W) (a), the effect of temperature and power at a constant time (7 min) (b), and the effect of time and power at constant temperature (70 °C) (c).

**Figure 5:**
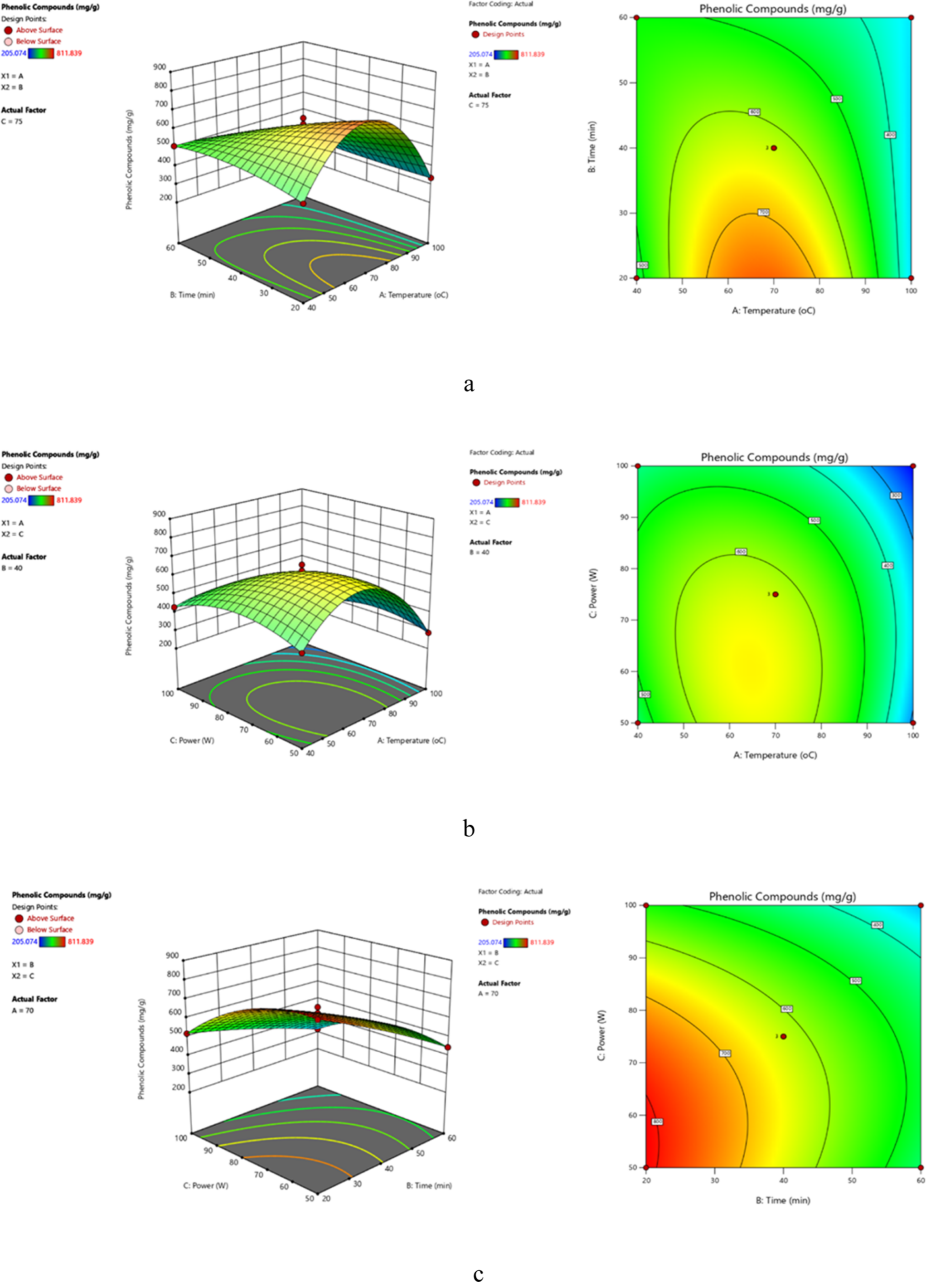
Three-dimensional and contour response surface plots of total phenolic compounds showing the effects of temperature and time at a constant optimum power (800 W) (a), the effect of temperature and power at a constant time (7 min) (b), and the effect of time and power at constant temperature (70 °C) (c) in UAE.

When the effect of temperature (*A*) and time (*B*) on the extraction yield and phenolic compounds were examined at a constant ultrasonic power value (midpoint of the power value: 75%), it was noted that the maximum extraction yield and phenolic content were reached at extraction conditions of 70 °C and 40 min (Figures 4a and 5a). When the effects of temperature (*A*) and power (*C*) were examined at constant extraction time (midpoint of the extraction time: 40 min), higher extraction yields were observed at 100 °C and 50% power, whereas higher phenolic compounds were obtained at 70 °C and 75% power (Figures 4b and 5b).

When the effects of extraction time (*B*) and power (*C*) were examined at constant temperature (midpoint of the temperature: 70 °C), both extraction yield and phenolic compounds were observed to be maximized at 20 min and 50% power (Figures 4c and 5c).

The predictive equations for extraction yield and total phenolic content were evaluated using the second-order equation depicted in the equation below
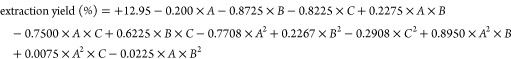

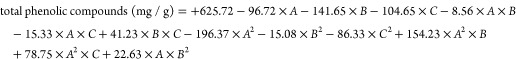


The UAE process takes place in two steps: transmission of ultrasonic waves and cavitation created in the dissolver by diffusion through herbal cell walls. In both steps, temperature and ultrasonic sound waves play critical roles.^33^ The optimum conditions for UAE of polyphenolic compounds from *P. brutia* bark were determined as 50% ultrasonic sound power and 20 min extraction time. The width and low wavelengths of ultrasonic sound waves play a crucial role in the extraction performance by shortening the time.^34^ In light of this information, it has been observed that the most suitable conditions for the breakdown of the analyte-matrix bonds of the sample and accordingly to obtain the phenolic compounds in high yield correspond to a power of 50% and a time of 20 min. When the sonic power is adjusted to 100%, the yield of the phenolic content is reduced, regardless of the extraction time. When the ultrasonic power exceeds 50%, the decrease in the amount of phenolic content can be interpreted as the adherence of phenolic components to the pulverized surface of the pine bark. UAE maximizes the extraction efficiency through enhanced mass transfer as well as increased diffusion by cavitation forces where bubbles in solid/liquid extraction can explosively collapse and create localized pressure, causing the breaking of plant tissue and improving the exhaust of intracellular substances into the cell.^35^ With the hydration of the pectinous material in the middle lamella of the plant tissue, the cell wall swells and softens, and the plant tissue is fragmented with high sonic waves.^41^ UAE is one of the cheapest, simple, fast, and effective techniques compared to traditional extraction processes, with the advantages of high repeatability, easy adjustment of parameters, low temperature, low solvent consumption, and lower energy input in relatively short times. Therefore, UAE technology is frequently used in the food, chemical, and material industry.^36^

In addition to the experiments conducted, a final UAE was carried out under the optimized conditions in order to verify the predictive capacity of the model. The optimum extraction conditions of 72 °C, 20 min, and 50% power in the UAE process yielded an extraction efficiency of 15.21%. When the UAE was repeated under the optimized conditions, a notable extraction yield of 15.20% was obtained. As for the total phenolic content, the optimum extraction conditions were determined as a temperature of 69.8 °C, a time of 20 min, and a power of 51%, yielding a phenolic content of 812.378 mg/g. When UAE was repeated under these conditions, a phenolic yield of 811.839 mg/g was achieved. Both results showed that there was no significant difference (*p* > 0.05) between the predicted and experimental values, validating the experimental conditions and the model.

### 3.3 UPLC Analyses of Extracts Obtained under Optimum Conditions

**Figure 6:**
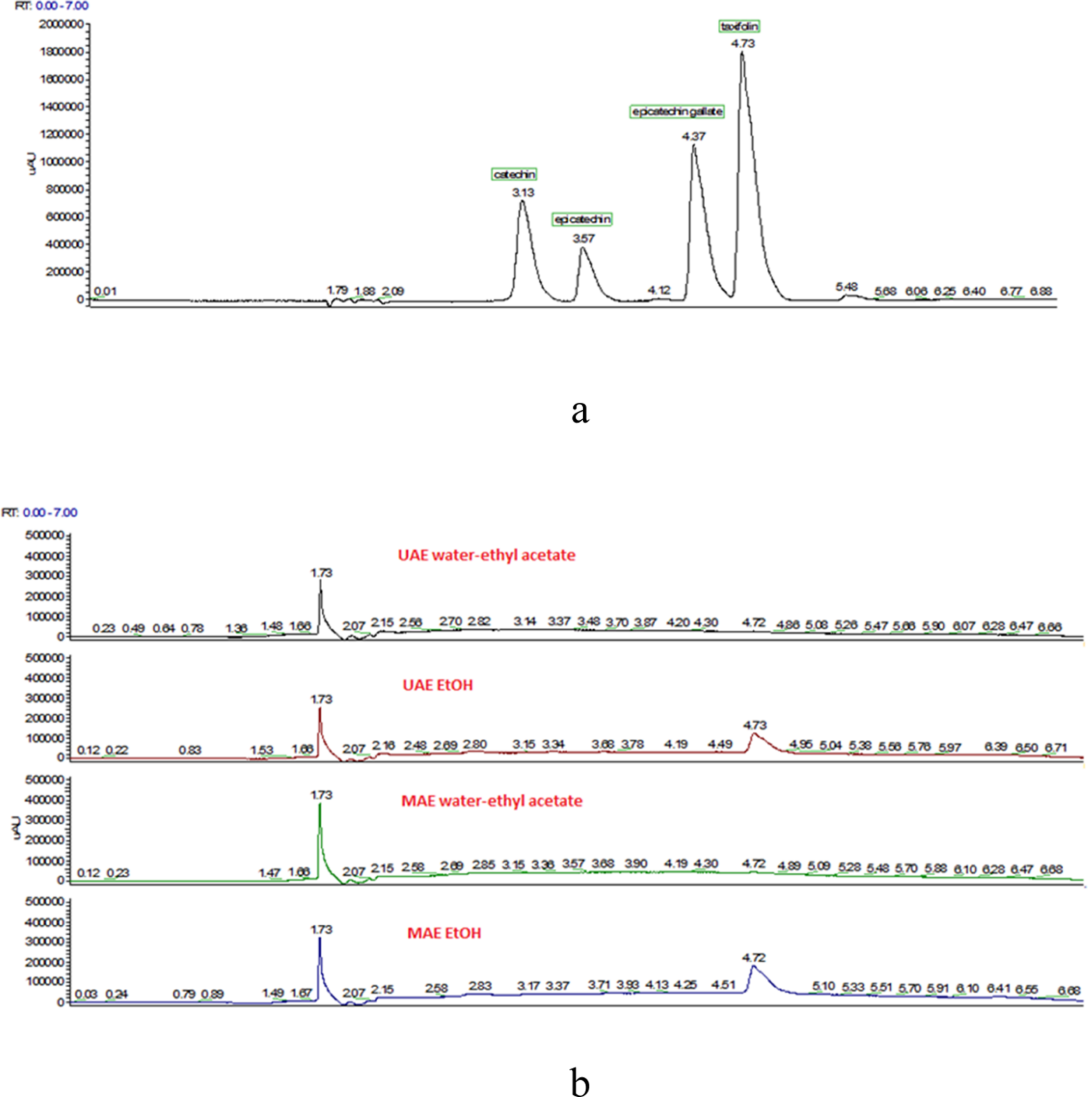
UPLC chromatograms of the standards of catechin, epicatechin, epicatechin gallate, and taxifolin. (a) UAE water–ethyl acetate and ethanol extracts obtained at 70 °C, 20 min, 50% power, and MAE water–ethyl acetate and ethanol extracts obtained at 70 °C, 10 min, and 900 W, respectively (b).

In the study, the amounts of taxifolin, (−)-catechin, (−)-epicatechin, and (−)-epicatechin gallate contents in the extracts obtained under optimum conditions were determined by ultra performance liquid chromatography (UPLC) device. UPLC chromatograms of catechin, epicatechin, epicatechin gallate, taxifolin standards as well as UAE and MAE extracts (water/ethyl acetate and ethyl alcohol) are depicted in Figure 6a. According to the results obtained, taxifolin and (−)-catechin were quantified in the extracts, but (−)-epicatechin and (−)-epicatechin gallate could not be detected. As seen in Table 5, when ethanol is used as a solvent, the taxifolin in MAE is 34.0 mg/g extract, whereas it is 23.5 mg/g extract in UAE. When water/ethyl acetate was used as the solvent, the amounts of catechin and taxifolin were acquired in much lower amounts. The solvating power of ethanol as a solvent here can be explained as increasing the solubility of phenolic compounds with the increase of hydrogen bonds and dipole–dipole interactions.^37^ Solvent type is the most important parameter influencing the efficacy of the extraction. Due to the polar nature of polyphenols, they can be easily dissolved in polar environments such as hydroalcoholic solvents. By changing the alcohol concentration in the mixture, phenolic fractions can be obtained much more easily by adjusting the polarity of the medium.^38,39^ The fact that the use of ethanol as an extraction solution increased the release of phenolic compounds from pine bark showed that the type of solvent used as a function of the extraction time was important. However, Figure 6b shows that catechin contents do not show significant differences when compared with extraction techniques and solvent type, except for MAE water/ethyl acetate extract. The results show that *P. brutia* is rich in taxifolin and catechin compounds. Studies examining the content of phenolic compounds indicate that *P. brutia* bark extracts contain more (−)-catechin and taxifolin than Pycnogenols.^19^*P. brutia* is a rich source of catechin and taxifolin.

**Table 5:** DPPH Radical Scavenging Activities of MAE, UAE, and Soxhlet Water–Ethyl Acetate along with Ethanol Extracts and Contents of Catechin and Taxifolin (mg/g of extract)

extraction method	extraction conditions	solvent type	DPPH %	catechin (mg/g extract)	taxifolin (mg/g extract)
UAE	70 °C, 20 min, 50% power	water/ethyl acetate	90.8	0.99	0.73
MAE	70 °C, 10 min, 900 W	water/ethyl acetate	86.2	ND^a^	1.58
Soxhlet	100 °C, 4 h	water/ethyl acetate	87.6		
UAE	70 °C, 20 min, 50% power	ethanol	82.9	0.81	23.5
MAE	70 °C, 10 min, 900 W	ethanol	71.1	1.05	34.0
Soxhlet	100 °C, 4 h	ethanol	81.9		

a ND: not detected.

### 3.4 Biological Activities of Extracts Obtained under Optimum Conditions

**Figure 7:**
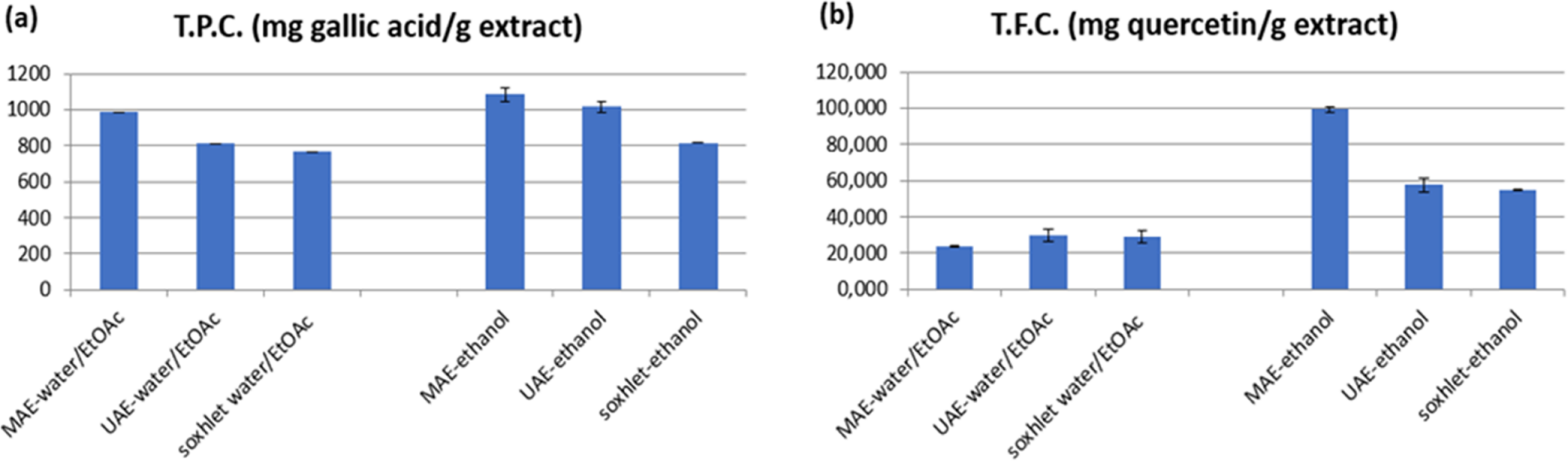
Total phenolic (a) and total flavonoid contents (b) of *P. brutia* bark extracts.

Polyphenolic compounds are known to have many health benefits, including anti-inflammatory, antidiabetic, antiallergic, antimicrobial, antiviral, antipathogenic, and antithrombotic.^40^ Solvents such as ethanol, methanol, acetone, and ethyl ester are generally used to obtain phenolic compounds.^37,41^*P. brutia* bark extracts contain polyphenolic compounds such as proanthocyanidins, phenolic acids, taxifolin, catechin, and epicatechin. Each of these compounds has antioxidant properties strong enough to protect cells from the damaging effects of reactive oxygen species known as free radicals.^42−44^ It has also been shown that Pycnogenol, a nutritional supplement prepared from French maritime pine bark extract can be used for photoprotection, reducing hyperpigmentation in human skin and improving the skin epithelial barrier role.^45^ As seen in Figure 7a, the phenolic contents of the extracts under optimum conditions determined for MAE, UAE, and Soxhlet extraction were examined. The highest phenolic content of 1083.51 mg/g was obtained by MAE performed at 70 °C temperature, 10 min, 900-W power, and 1:10 solid–liquid (ethanol) ratio (g/mL), which is similar to the content of UAE ethanol extracts (1014.8 mg/g), while comparatively lower values (813.9 mg/g) were obtained for Soxhlet ethanol extract. However, when water/ethyl acetate was used as a solvent, the phenol content (987.3 mg/g) was found to be the highest in the extracts obtained with MAE. The phenol contents of the extracts obtained by UAE and Soxhlet extractions were 811.9 mg/g and 766.4 mg/g, respectively. In addition, Yesil-Celiktas carried out water extraction of *P. brutia* bark and determined the phenol compound as 936.60 mg gallic acid/g extract.^8,17^ In this respect, *P. brutia* is a beneficial resource of phenolic compounds. The results of the total polyphenol determination showed that MAE and UAE are much more effective techniques than Soxhlet extraction. MAE is gaining popularity due to its advantages such as less time-consuming, less solvent usage, control of related parameters, and better improvement. This technique is remarkably productive in point of efficiency, extraction time, and microwave energy consumption than traditional techniques.^38^ Compounds that constitute an important group of natural antioxidants are flavonoids.^46^ Polyphenolic compounds such as flavonoids, anthocyanins, and catechins are the main components that give taste and color to fruits and vegetables, and thanks to their capacity to inhibit free radicals, they have antioxidative, anti-inflammatory, antiallergic, antimicrobial and It has chemical and biological activities including many therapeutic effects such as anticancer.^47−49^ It is also seen in the results obtained that the *P. brutia* extracts used in the study are quite rich in terms of the flavonoid component content. Among the three different extraction techniques in the study, the highest total flavonoid concentration (99.4 mg/g) was obtained using the MAE method when ethanol was used as the solvent. The lowest flavonoid content (55.1 mg/g) was obtained in Soxhlet extraction with ethanol. In addition, when the flavonoid contents in the water/ethyl acetate extracts are examined in Figure 7b, the results of the three extraction techniques were observed to be close to each other. Total flavonoid contents in MAE, UAE, and Soxhlet water–ethyl acetate extracts were quite low compared to ethanol extracts, and found to be 23.7, 30.1, and 28.8 mg/g, respectively. Thus, ethanol was elicited as the most suitable solvent for obtaining a high amount of flavonoid compounds from *P. brutia*. In a similar study, *P. brutia* was extracted using various solvents (acetate, *n*-hexane, ethyl ethanol, and dichloromethane), and the highest flavonoid content was also obtained with ethanol.^17^ The use of ethanol as a solvent increases the polarity, and as a result, hydrogen bonds and dipole–dipole interactions increase, so that many more phenolic compounds can be obtained.^39^ The results showed that the solvent and the extraction technique are important parameters with respect to the flavonoid content.

Cells in the human body suffer permanent damage over time from free radicals, which is an inevitable outcome of life. Free radicals, formed by the breakdown of oxygen molecules, can occur even through breathing, as well as under environmental factors such as air pollution, malnutrition, and stress. Oxidative damage caused by free radicals can lead to different levels of cell and tissue damage.^50,51^ Antioxidants are molecules generally responsible for preventing the formation of free radicals, neutralizing the resulting radicals, and eliminating damaged molecules.^52,53^ Antioxidants, especially abundant in *Pinus* spp., contribute to the continuity of vital activities against oxidative damage.^54,55^ Free radical scavenging activities of *P. brutia* bark extracts obtained by three different extraction techniques were determined using a DPPH radical scavenging assay. It was seen that red pine bark had the highest free radical scavenging activity (86.4%) among different pine species in another study.^17^ Although total phenolic and flavonoid contents were maximized with ethanol was employed as the extraction solvent, higher DPPH free radical scavenging values were obtained (UAE: 90.8%, MAE: 86.2%, Soxhlet: 87.6%) when water ethyl acetate was used as a solvent (Table 5). However, MAE with water–ethyl acetate can be preferred due to shorter extraction times and high repeatability due to the ease of control of process parameters.

### 3.5 Catalytic and Noncatalytic Steam Gasification of Pine Bark Residues

**Figure 8:**
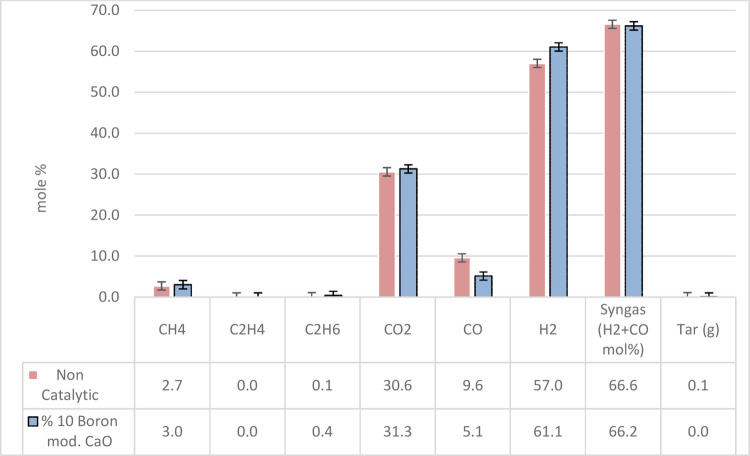
Gas composition, syngas, and tar amount of noncatalytic and catalytic steam gasification of pine bark.

Gas composition (mol %), syngas (H_2_ + CO mol %), and tar amount (g) of noncatalytic and catalytic steam gasification of pine bark are shown in Figure 8. The highest hydrogen content of 60.8 mol % was achieved by utilizing a 10% boron-modified CaO alkali catalyst. However, minor decreases were observed in CO and total syngas compositions, while CO_2_ content slightly increased compared to noncatalytic gasification. The reason is that between 500 and 850 °C, endothermic reactions dominate. After steam reforming of tar at a higher temperature, carbon dioxide, and hydrogen are produced with the reaction of carbon monoxide and steam.^36^ The addition of a 10% boron-modified CaO alkali catalyst resulted in a slight increase in CH_4_, while it yielded a higher increase in the C_2_H_4_ content. For both gasifications, the C_2_H_6_ content was not affected, while great tar reduction was achieved by catalytic gasification. To enhance the yield of noncondensable gases such as H_2_, CO, CO_2_, and CH_4_, CaO is widely used for tar cracking purposes.^56^ Based on the findings of this study, the introduction of a 10% boron modification to the CaO alkali catalyst has been found to augment tar cracking, resulting in an elevated hydrogen concentration.

Gasifying agents have a significant effect on gas composition. In the study conducted to investigate synthesis gas formation, CO_2_-assisted gasification of pine bark was carried out at varying temperatures.^57^ The results showed that with increasing temperature, CO_2_ enriched the CO content and decreased the H_2_ content. In another study, biomass gasification was undertaken using air as the gasifying agent, focusing specifically on biomass species, namely, spruce, alder, and pine. The gasification process involved varying temperatures (750, 850, and 950 °C). For various wood biomass species, carbon monoxide (CO) dominated combustible gases, ranging from 9.3 to 10.4% at 750 °C, 14.7 to 15.9% at 850 °C, and 19.9 to 21.4% at 950 °C. Hydrogen (H_2_) showed varying concentrations: 0.7–1.3% at 750 °C, 2.4–2.5% at 850 °C, and 4.0–4.3% at 950 °C.^58^ In contrast to prior investigations, both the employed catalyst and gasifying agents exhibited a notable impact on the composition of the hydrogen-rich gas in the current study.

### 3.6 Activated Carbon through Noncatalytic and Catalytic Steam Gasification

BET surface area and total pores are shown in Table 6. According to the results, PB-B exhibited higher surface area and pore volume (1358.32 m^2^/g and 1.05 cm^3^/g) than PB (437.81 m^2^/g and 0.36 cm^3^/g). PB-B showed 0.31 cm^3^/g micropore volume and 0.74 cm^3^/g mesopore volume. The activated carbon derived from date seeds employing diverse chemical agents was fabricated while varying the chemical-to-biomass impregnation ratio (5:1, 4:1, 3:1, 2:1, 1:1, 1:0.5).^59^ The activation process involved a range of temperatures at 600, 700, 800, and 900 °C. The highest BET surface areas of 912 and 577 m^2^/g were achieved by 5:1 impregnation ratio of KOH and 2:1 impregnation ratio of H_2_SO_4_ at 900 °C, respectively. Compared with chemical activation, PB and PB-B materials produced via steam activation exhibited high surface area and porous volume.^60^ While the chemical activation method exhibits certain advantages over the physical activation approach, it comes with certain limitations.

**Table 6:** BET Surface Area and Porosity Results of Activated Carbons

sample	*S*_BET_ (m^2^/g)	*V*_T_ (cm^3^/g)	*S*_micro_ (m^2^/g)	*V*_micro_ (cm^3^/g)	*S*_meso_ (m^2^/g)	*V*_meso_ (cm^3^/g)	*D*_av_ (Å)
PB^a^	437.81	0.36	186.58	0.08	251.23	0.28	37.59
PB-B^b^	1358.32	1.05	652.39	0.31	705.93	0.74	34.04

a PB: Pine Bark derived activated carbon that was produced through non-catalytic steam gasification.

b PB-B: Pine bark-derived activated carbon that was produced through catalytic steam gasification.

**Figure 9:**
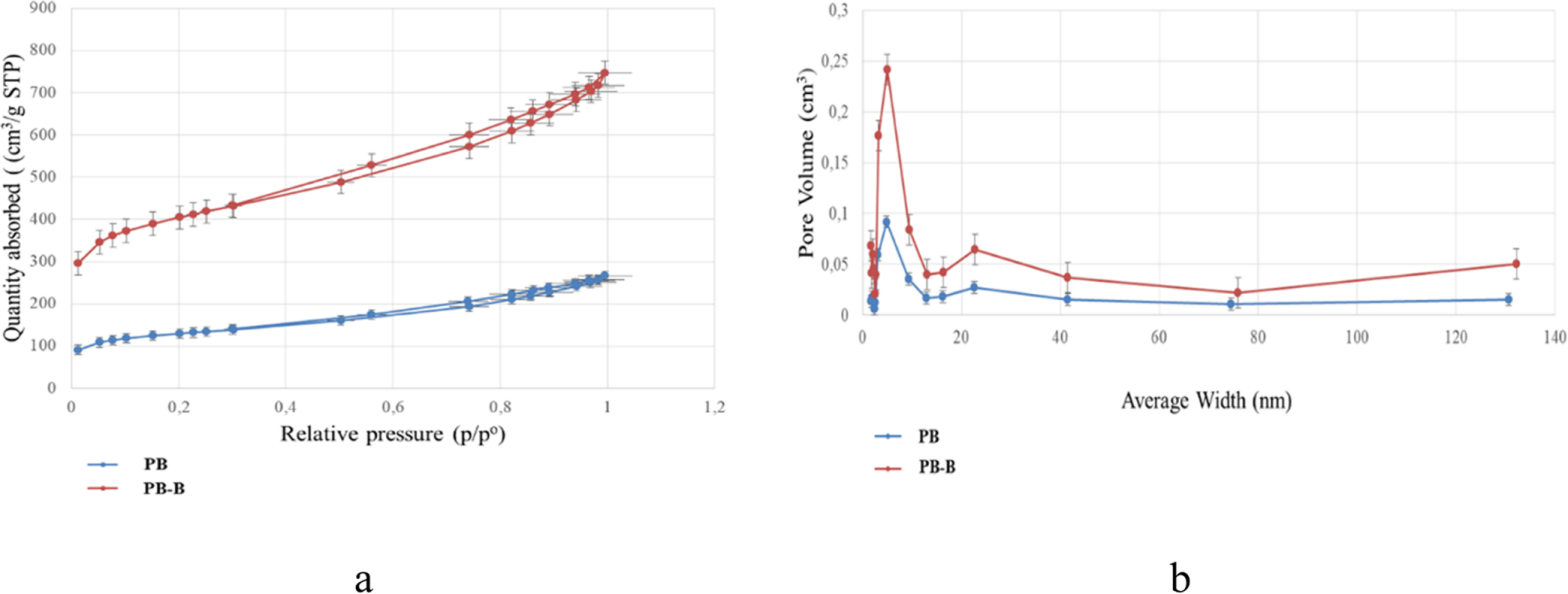
N_2_ adsorption–desorption isotherms of activated carbons (a) and pore size distribution of activated carbons (b).

These include the need to eliminate inorganic impurities from activated carbon through a washing process and the corrosive effects of the activating agents.^61,62^ Despite the incorporation of a catalyst in this work, it was not directly blended with *P. brutia*. Activated carbon was instead derived through steam gasification. The utilized catalyst was subsequently recovered postprocess, and the resulting activated carbon obviated the necessity for any purification steps owing to its indirect interaction with the catalyst. Consequently, the production of PB-P was accomplished through an environmentally friendly approach.^62^ N_2_ adsorption–desorption isotherms of the activated carbons were exhibited in Figure 9a. According to the International Union of Pure and Applied Chemistry (IUPAC) physiosorption isotherm classifications, two activated carbons displayed IV-type isotherms, signifying the presence of a mesoporous structure.^63^ Pore size distributions of activated carbons are shown in Figure 9b. Pore diameter in PB is almost distributed between 3 and 9 nm and showed a very sharp peak in this range. PB-B showed a very sharp peak between 2 and 16 nm, mainly concentrated on 2 and 5 nm. In contrast to PB, PB-B exhibited micropores that are mostly concentrated on 1 and 2 nm, and also mesopore structure within 2–5 nm. The micropore structure offers a substantial effective internal surface area conducive to the adsorption of electrolyte ions.

Simultaneously, the pore structure featuring diameters in the range of 2–4 nm establishes a pathway of low resistance for efficient ion transport into the inner surface area.^64^ Due to its porous structure, PB-B can be used as an electrode material in supercapacitor applications.

## 4 Conclusions

In this study, *P. brutia* bark was extracted under different conditions (MAE water–ethyl acetate 987.3 mg/g extract, UAE water–ethyl acetate 811.9 mg/g extract, Soxhlet water–ethyl acetate 766.4 mg/g extract, MAE ethanol 1083.5 mg/g extract, UAE ethanol 1014.8 mg/g extract, Soxhlet ethanol 813.9 mg/g extract) yielding high concentrations of total phenolic components. The rich phenolic content of *P. brutia* bark extracts increases the utilization potential in food, pharmaceutical, and biomedical industries. Furthermore, catalytic and noncatalytic steam gasification of pine bark residue yielded 57.3 and 60.8 mol % H_2_, respectively. In addition, excellent tar reduction was achieved by utilizing a 10% boron-modified CaO alkali catalyst. The obtained activated carbon (PB-B) exhibited 1358.32 m^2^/g BET surface area and 1.05 cm^3^/g total pore volume, which has potential use as an adsorbent for removing heavy metals and electrode material for supercapacitor applications. Such a holistic utilization of pine bark is envisaged to promote sustainable development and contribute to a circular economy through an integrated biorefinery approach.
